# Context-dependent cGAS–STING signaling in inflammatory bowel disease: cell specificity, lesion state, and therapeutic windows

**DOI:** 10.3389/fimmu.2026.1970206

**Published:** 2026-10-20

**Authors:** Huihui Bian, Wenjing Jin, Yuhong Chen, Li Su, Rongsheng Zhang

**Affiliations:** 1 Institute of Translational Medicine, Shanghai University, Shanghai, China; 2 School of Medicine, Shanghai University, Shanghai, China; 3 Department of Hepatobiliary Surgery, The Affiliated Wuxi People’s Hospital of Nanjing Medical University, Wuxi, China

**Keywords:** cGAS-STING pathway, inflammatory bowel disease, innate immunity, mucosal immunity, therapeutic targeting

## Abstract

Whether cyclic GMP–AMP synthase (cGAS)–stimulator of interferon genes (STING) signaling should be inhibited in inflammatory bowel disease (IBD) cannot be answered from pathway abundance alone. Canonical ER–Golgi–TBK1–IRF3/NF-κB signaling can amplify intestinal inflammation, whereas nuclear STING–AHR signaling, T-cell-intrinsic STING, ILC3 sensing, and epithelial antimicrobial programs may support microbial tolerance, host defense, or repair. We therefore examine STING output across subcellular location, signal source and duration, responding cell type, lesion state, and strength of evidence. The most consistent pathogenic evidence comes from experimental colitis with sustained DNA exposure and myeloid or epithelial stress; direct human evidence is more limited, mainly cross-sectional, and heterogeneous between ulcerative colitis and Crohn’s disease. This distinction limits inferences from increased cGAS/STING expression or interferon-stimulated genes, which do not establish cGAMP production, STING trafficking or phosphorylation, TBK1–IRF3 activation, or a causal biological outcome. The available data support a working model rather than a validated temporal sequence: protective and pathogenic programs can coexist within the same lesion, and broad systemic inhibition may impair antimicrobial defense, tolerance, mucosal repair, or tumor immune surveillance. Translation will therefore require cell-resolved target-engagement biomarkers and locally delivered, time-limited interventions tested in longitudinal human tissue and models that preserve microbiota and lesion heterogeneity.

## 1 Introduction

### 1.1 The STING paradox is a compartment- and lesion-state problem

Inflammatory bowel disease (IBD) is sustained by a damaged mucosal surface in which epithelial injury, microbial imbalance, and immune activation reinforce one another (1, 2). Current biologics and small molecules have changed clinical practice, but most act after inflammatory circuits are established (3). They do not fully explain why upstream danger sensing becomes persistent in some patients, why repair remains incomplete, or why relapse follows apparently successful inflammatory control.

The cGAS–STING pathway sits at this unresolved interface. It detects misplaced host or microbial DNA, responds to bacterial cyclic dinucleotides, and links mitochondrial stress and barrier failure to type I interferon and inflammatory cytokine programs (4, 5). STING also has non-canonical functions outside the familiar ER–Golgi signaling route. The resulting literature is difficult to reconcile because a pathway involved in damage sensing, antimicrobial defense, tolerance, and repair cannot be reduced to a single inflammatory switch.

### 1.2 Current therapies leave the upstream regulation problem unresolved

The IBD armamentarium now includes anti-tumor necrosis factor (TNF) agents, anti-integrin antibodies, anti-interleukin-23 biologics, Janus kinase inhibitors, and sphingosine-1-phosphate receptor modulators (6–9). Yet primary non-response, secondary loss of response, relapse after withdrawal, and safety trade-offs remain familiar problems. These limitations do not argue against current therapy; they show that durable control also depends on understanding how epithelial damage, microbial signals, and innate sensing become locked into chronic mucosal inflammation.

### 1.3 The central question: when is STING protective, and when is it pathogenic?

In IBD, a STING signal is interpretable only when its cellular and subcellular location, timing, intensity, and biological consequence are defined (10, 11). Danger signals provide the input, mucosal compartments constrain the response, and lesion state determines whether the output favors surveillance, tolerance, inflammatory amplification, epithelial injury, or repair. The clinically relevant problem is therefore not whether STING is globally beneficial or harmful, but whether a defined lesion contains a pathogenic, targetable STING program without concurrent dependence on protective STING functions.

Previous reviews have summarized the bidirectional effects of cGAS–STING in intestinal inflammation and colitis-associated cancer (12, 13). The present Review addresses a narrower unresolved problem: how evidence directness, subcellular localization, cell identity, lesion state, and pharmacological mechanism should jointly constrain interpretation. This two-axis framework distinguishes the source of evidence from the depth to which pathway activation or target engagement was assessed, and uses this distinction to evaluate therapeutic claims.

### 1.4 Literature search strategy

A structured literature search was conducted in PubMed and the Web of Science Core Collection from database inception through September 23, 2026. The search combined pathway-related terms with disease-related terms using the following core Boolean strategy: (cGAS OR STING OR TMEM173 OR cGAMP) AND (“inflammatory bowel disease” OR “ulcerative colitis” OR “Crohn’s disease” OR colitis OR “intestinal inflammation”). The search syntax was adapted to the indexing requirements of each database; in Web of Science, these terms were searched within the Topic field. Reference lists of included primary studies and relevant review articles were also screened to identify additional publications. The final searches were conducted on September 23, 2026.

Peer-reviewed primary studies were prioritized when they examined human IBD tissue or blood, experimental colitis, intestinal organoids or primary intestinal cells, or cell-specific genetic or pharmacological perturbation of cGAS–STING. Titles and abstracts were screened for disease and pathway relevance, followed by full-text assessment of the experimental system, cell type, subcellular localization, target-engagement readout, and biological outcome. Duplicate records were identified by DOI, PMID, or exact title. Conference abstracts, protocols, reports without a cGAS/STING-specific readout, and non-intestinal studies without a transferable mechanism were excluded. Non-IBD studies were retained only when they established a mechanism or pharmacological principle not yet tested directly in intestinal inflammation; these are identified as extrapolated evidence. Reviews were used for background and source discovery, not as sole support for mechanistic claims.

Evidence was assessed on two independent axes. The first records source and directness: direct human IBD evidence, causal evidence from experimental colitis, mechanistic intestinal evidence, or extrapolated evidence from non-IBD systems. The second records activation depth: component abundance, cGAMP production, STING localization or phosphorylation, downstream TBK1–IRF3/NF-κB/type I interferon output, and a linked cellular or tissue phenotype. Confidence was increased by target-specific perturbation, orthogonal activation readouts, replication across models, and concordant human localization, and reduced by expression-only evidence, single-model dependence, or extrapolation. Because this was a narrative synthesis, records were not enumerated in a PRISMA flow diagram and no formal risk-of-bias or GRADE score was assigned (**Table 1**).

**Table 1:** Translational evidence framework for cGAS-STING signaling in IBD.

Evidence source	Representative systems	Establishes	Required evidence	Confidence/limits
Direct human IBD	Biopsy, blood, single-cell, spatial, or organoid-linked human studies (14–24)	Relevance, localization and association	cGAMP; STING localization or phosphorylation; downstream signaling; functional readouts	Moderate for replicated, cell-resolved relevance; low for causality without perturbation
Experimental colitis	Genetic, pharmacological, or microbiota perturbation in experimental colitis (10, 11, 16, 18–21, 25–43)	Causality in the tested model, cell type and time window	Target-specific perturbation, engagement and a linked biological outcome	High within replicated models; transfer to human IBD remains limited
Mechanistic intestinal models	Organoids, primary epithelial or immune cells, and co-cultures (10, 16–20, 25, 28–34, 36–39, 42, 44–47)	Cell-intrinsic mechanism and pathway order	Orthogonal readouts with rescue, epistasis or loss-of-function	Moderate when convergent; limited tissue and microbial context
Extrapolated mechanisms	Non-IBD disease models or non-intestinal systems (45, 48–56)	Biological plausibility or pharmacological principle	Direct engagement does not establish intestinal relevance	Low for IBD until reproduced in intestinal models
Translational pharmacology	Representative modulators in **Table 3**	Feasibility, exposure and druggability	Engagement, pharmacokinetics, efficacy, cell/tissue selectivity and safety	Low for clinical use without human IBD engagement and chronic safety data

Interpretation rule: evidence source and activation depth are independent. A non-IBD study may show direct molecular engagement but still provide weak IBD-specific evidence; a human biopsy may be disease-proximal but remain expression-only and non-causal.

## 2 Inputs that set the mucosal STING activation threshold

### 2.1 Canonical and nuclear STING outputs

In the canonical pathway, cytosolic double-stranded DNA activates cGAS, which generates 2′3′-cyclic GMP–AMP (cGAMP). cGAMP then binds ER-resident STING. Ligand-bound STING oligomerizes and traffics from the ER to the Golgi, where it recruits TANK-binding kinase 1 (TBK1) to phosphorylate interferon regulatory factor 3 (IRF3), while also engaging nuclear factor κB (NF-κB)-linked programs (57–59). These events should be distinguished experimentally from pathway-component abundance: cGAMP production, STING redistribution and phosphorylation, phospho-TBK1/phospho-IRF3, NF-κB activation, and type I interferon output report different levels of pathway activity.

STING also has a nuclear function that is not captured by the canonical ER–Golgi model. Nuclear STING1 can bind and activate the aryl hydrocarbon receptor (AHR), thereby shaping commensal composition and intestinal homeostasis; this program is independent of DNA sensing and competes functionally with cytoplasmic cGAS–STING signaling (25). Subcellular localization is therefore a determinant of pathway output rather than a descriptive detail. Human transcriptomic and single-cell studies establish the presence of pathway components and interferon-related niches in inflamed mucosa, but do not show which STING pool is responsible (14, 15).

DEAH-box helicase 9 (DHX9) further illustrates this branch specificity. In the nuclear STING1–AHR system, DHX9 acts as a negative regulator of AHR activation (25). In intestinal stem cells, by contrast, DHX9 restrains R-loop accumulation and genomic instability; epithelial DHX9 loss permits R-loop accumulation and genomic instability, thereby driving aberrant cGAS–STING activation, impairing stem-cell function, and increasing susceptibility to experimental colitis (16). These findings are compatible because they concern different STING pools and cellular compartments. Compensatory activation of nuclear STING1–AHR signaling after DHX9 loss has not been demonstrated in colitis and should remain a testable hypothesis.

### 2.2 Epithelial injury as an endogenous source of DNA danger signals

Epithelial injury changes both the source and the biological consequences of STING activation. Repeated inflammatory stress disrupts tight junctions and increases epithelial cell death, releasing mitochondrial DNA, nuclear DNA fragments, and extracellular vesicle-associated dsDNA into the local microenvironment (17). These damage-associated molecular patterns can be taken up by neighboring myeloid cells and activate cGAS-STING signaling. In experimental colitis, epithelial injury-derived TFAM-mtDNA complexes have been linked to dendritic-cell STING-IL-12 activation, Th1/Th17 skewing, and aggravated inflammation (10). Oxidative stress and mitochondrial dysfunction can further amplify mitochondrial DNA leakage, creating a feedback loop between epithelial injury, DNA release, STING activation, and inflammatory amplification (60).

### 2.3 Microbial inputs: infection, dysbiosis, and tolerance signals

STING can respond to microbial inputs through at least two routes. Microbial DNA that reaches the cytosol can be recognized by cGAS, whereas bacterial cyclic dinucleotides can bind STING directly (5, 61). These direct agonists are mechanistically distinct from metabolites that alter epithelial integrity, mitochondrial stress, autophagy, AHR activity, or the inflammatory threshold without binding STING.

IBD is usually not initiated as an acute infection (62). In this chronic inflammatory setting, pathogen-associated molecular patterns (PAMPs) more often act through pre-existing barrier defects, dysbiosis, and local immune imbalance (62, 63). Their contribution is therefore best viewed as amplification of an already vulnerable mucosal network rather than as a single initiating event.

Persistent exogenous stimulation in IBD is better conceptualized in terms of dysbiosis and barrier leakage than acute infection. Barrier leakage can increase exposure to microbial DNA and cyclic dinucleotides, while microbial community structure influences the metabolites that condition epithelial and immune responses. Dysbiosis promotes STING ubiquitination and accumulation in myeloid cells in experimental colitis (26), and microbiota-dependent STING activation is constrained by OTUD3 in a recent UC model (27). These data support a microbiota–STING interaction, but do not establish a single taxon or metabolite as a universal driver across UC and CD.

Microbiota-associated STING signaling is not invariably inflammatory. ILC3-intrinsic STING permits direct microbial sensing, CCR7-dependent migration, and induction of microbiota-specific regulatory T cells in a Helicobacter hepaticus model (28). This direct STING-dependent finding is distinct from the broader observation that ILC3s select microbiota-specific regulatory T cells (29). Inflammation-induced STING hyperactivation caused ILC3 death in the former study; the less specific term “exhaustion” is therefore avoided. cGAMP-associated metabolic programming has also been linked to intestinal immune tolerance (30).

### 2.4 The microbiota–metabolite interface

Microbial metabolites should be separated from microbial STING agonists. Butyrate is not a direct STING ligand, yet it has been reported to suppress cGAS–STING–NLRP3-associated epithelial pyroptosis in Crohn’s disease-derived systems and to limit STING-dependent ER stress and CD4+ tissue-resident memory T-cell accumulation in a dextran sulfate sodium (DSS) colitis model (31, 32). Tryptophan-derived indoles, including indole-3-ethanol, indole-3-pyruvate, indole-3-aldehyde, and indole-3-acetic acid (IAA), can engage AHR or related barrier programs and thereby converge functionally with nuclear STING–AHR signaling without directly activating STING (33, 44).

The direction of this interaction may also run from host DNA sensing to microbial metabolism. In a 2026 study of diet-induced obesity in male mice, intestinal epithelial cGAS–STING–type I interferon signaling reduced *Lactobacillus murinus* abundance and IAA production (45). This provides mechanistic evidence for an epithelial cGAS–STING–microbiota–metabolite axis, but it is not an IBD study and should not be treated as direct evidence for colitis. Secondary bile acids and polyamines may likewise influence STING indirectly through epithelial stress, mitochondrial function, autophagy, or immune-cell metabolism; direct target engagement has not been established. The practical distinction is therefore between microbial DNA or cyclic dinucleotides that can engage cGAS–STING directly and metabolites that modify the tissue state in which STING operates.

### 2.5 Crosstalk with inflammasomes, RNA sensing, autophagy, and cell death

cGAS–STING operates within a network of pattern-recognition and stress pathways rather than in isolation. TLR signaling can prime cytokine and inflammasome components; NLRP3 can convert mitochondrial stress and ionic disturbance into caspase-1 activation, gasdermin D cleavage, and pyroptosis; AIM2 can respond directly to cytosolic double-stranded DNA; and MAVS/RIG-I pathways couple RNA sensing to overlapping interferon outputs. Autophagy can either remove damaged mitochondria and terminate STING activity or, when impaired, permit persistent DNA exposure and epithelial stress (31, 63–65).

Attribution therefore requires pathway-specific evidence. cGAS dependence, cGAMP generation, STING trafficking or phosphorylation, and loss of the response after STING perturbation support a STING-mediated mechanism. ASC specks, caspase-1 activation, gasdermin D cleavage, and interleukin-1β maturation define inflammasome engagement but do not by themselves distinguish NLRP3 from AIM2. Conversely, AIM2-dependent responses to cytosolic DNA should not be assigned to STING without direct perturbation (48). These interacting pathways may converge on pyroptosis or epithelial injury while remaining pharmacologically distinct.

### 2.6 From upstream inputs to cellular programs

Endogenous DNA released after epithelial injury, microbial DNA and cyclic dinucleotides, and metabolic or autophagic stress jointly set the probability and persistence of STING activation. They do not determine the biological outcome alone. The same input may support inflammatory amplification in macrophages, antigen-presenting programs in dendritic cells, tolerance or activation-induced death in ILC3s, and defense-to-injury transitions in intestinal epithelial cells. The next section therefore treats cellular and subcellular localization as the principal constraints on STING output (**Figure 1**).

**Figure 1:**
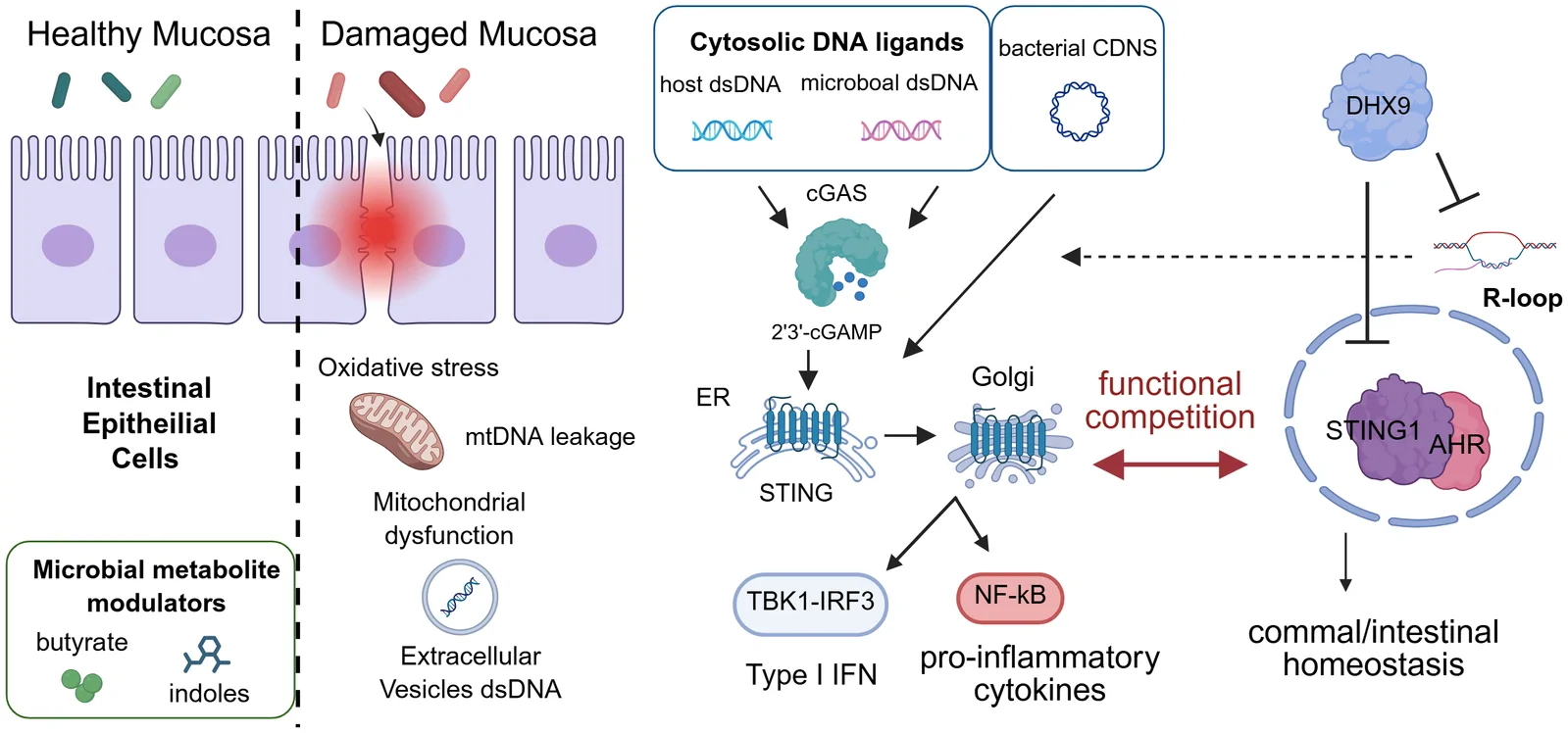
Compartment-specific STING signaling in injured intestinal mucosa. The schematic contrasts healthy and damaged intestinal epithelium. Barrier injury, oxidative stress and mitochondrial dysfunction release host nuclear and mitochondrial DNA, while microbial dsDNA and extracellular-vesicle-associated dsDNA add cytosolic ligands. cGAS-generated 2′3′-cGAMP and bacterial cyclic dinucleotides engage the canonical ER-to-Golgi STING route. The nuclear STING1–AHR program is a distinct, DNA-sensing-independent branch that competes functionally with canonical signaling. DHX9 inhibition of nuclear STING1–AHR activation and DHX9 restraint of R-loop-driven cGAS–STING activation in intestinal stem cells derive from distinct experimental contexts; their placement in one schematic does not imply a continuous pathway or compensatory nuclear STING1–AHR activation after DHX9 loss. Created in BioRender. Bian, H. (2026) https://BioRender.com/i8w58d2.

## 3 Mucosal compartments that shape STING output

### 3.1 Cell type is a major determinant of STING output

Cell specificity explains much of the apparent contradiction in studies of STING in IBD (12). The same upstream disturbance can elicit distinct downstream programs in macrophages, dendritic cells, epithelial cells, ILC3s, and regulatory immune networks. A macrophage-rich inflammatory lesion, an injured epithelial surface, and an ILC3-dependent tolerance niche are not interchangeable settings, even when STING-associated signals are detected in each compartment (**Figure 2**).

**Figure 2:**
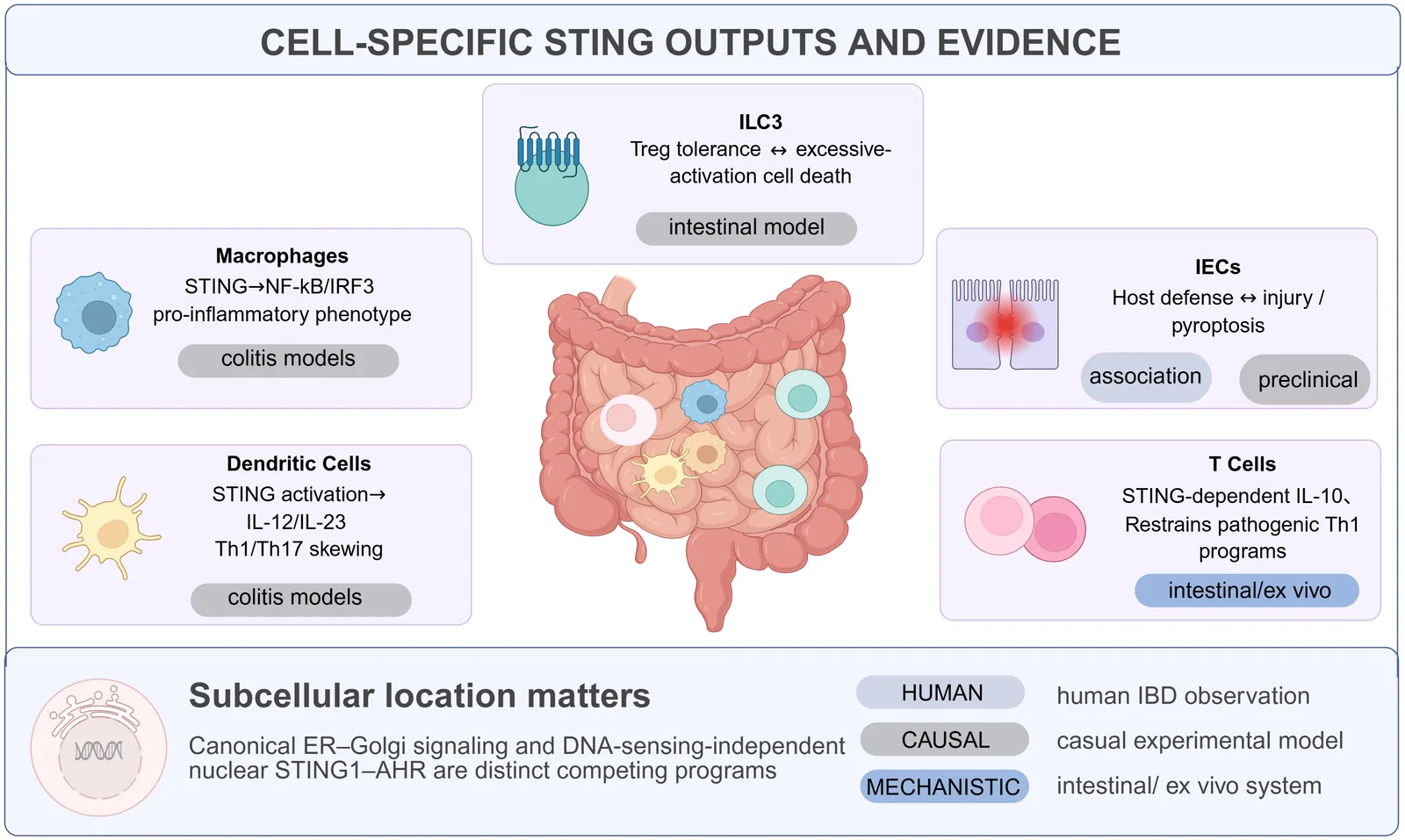
Cell-specific STING outputs and evidence in the intestinal mucosa. The schematic compares compartment-specific STING outputs and summarizes the dominant evidence associated with each mucosal compartment. Human observations, causal experimental evidence, and intestinal or ex vivo mechanistic evidence should be interpreted as separate evidence classes; descriptive labels within the schematic denote study context rather than a formal grading scale. Dendritic-cell evidence directly supports a STING–IL-12-associated Th1/Th17 response in experimental colitis, whereas broader IL-23 biology is not presented as direct intestinal STING causality. Created in BioRender. Bian, H. (2026) https://BioRender.com/x6056ep.

The evidence is also uneven across compartments. Myeloid cells provide the strongest link between STING and inflammatory amplification, whereas epithelial and lymphoid compartments show more conditional or model-dependent effects. This distinction matters therapeutically. A claim that STING is pathogenic in active colitis may be reasonable for selected macrophage or dendritic-cell programs, but it should not be extended automatically to epithelial repair, microbial tolerance, or regulatory circuits.

### 3.2 Macrophages: inflammatory amplification in active lesions

Myeloid STING is most relevant during active inflammatory amplification. Intestinal macrophages normally clear apoptotic material, preserve tolerance, and restrain excessive responses to commensal organisms (66). In inflammatory settings, persistent DNA exposure and microbial products may shift macrophages away from homeostatic functions and toward inflammatory cytokine production. Evidence from metabolic inflammation supports the biological plausibility of this transition but does not constitute direct IBD evidence (67).

Within experimental colitis, intestinal myeloid STING is best supported as an amplifier rather than an isolated trigger. Dysbiosis-dependent STING accumulation has been demonstrated in intestinal myeloid cells (26), and low-dose ganciclovir attenuated DSS-induced colitis and was associated with reduced STING signaling in colonic macrophages (34). By contrast, work on circulating microbial DNA-containing vesicles or macrophage cGAS activation in metabolic inflammation provides mechanistic plausibility but is extrapolated rather than direct IBD evidence (67).

For active, injury-rich experimental lesions, myeloid and cGAS–STING-associated inflammatory signaling provides a rationale for pathway inhibition (34–36). This inference is strongest for the specific experimental systems in which cell attribution or STING dependence was tested. It does not establish that macrophage STING is uniformly pathogenic in human UC or CD, and it does not exclude antimicrobial, regulatory, or repair-associated functions in other lesion states.

### 3.3 Dendritic cells: linking DNA sensing to adaptive immune skewing

Dendritic-cell STING can link epithelial injury to adaptive immune skewing in experimental colitis. TFAM-bound mitochondrial DNA released after epithelial damage activates a dendritic-cell STING–IL-12 axis and promotes Th1/Th17 inflammation (10). DNA-containing extracellular vesicles can also prime dendritic cells in non-IBD systems (49). The TREX1-deficient autoimmunity model cited here demonstrates that cGAS activation in conventional dendritic cells can drive systemic autoimmunity (50), but it is not an IBD study and is used only to support mechanistic plausibility.

The conclusion should therefore remain narrow: dendritic-cell STING can translate epithelial DNA damage into cytokine and antigen-presenting programs, but the directness of evidence differs between experimental colitis and non-intestinal immune models. Human lesions contain overlapping microbial, cytokine, and damage signals, so a dendritic-cell STING mechanism requires cell-resolved target-engagement data rather than inference from bulk interferon signatures.

### 3.4 T cells and Treg-related networks: adaptive immune balance

Adaptive effects should be divided into indirect and T-cell-intrinsic routes. Indirectly, STING activation in dendritic cells or macrophages can alter IL-12, IL-23, type I interferons, and co-stimulatory signals that shape Th1, Th17, and regulatory T-cell responses (10, 51). Such effects belong to an antigen-presenting-cell network and should not be attributed to T-cell STING.

Direct evidence also exists. In adoptive-transfer and DSS models, T-cell-intrinsic STING reprogrammed pathogenic Th1 cells toward IL-10 production through STAT3, Blimp-1, and mitochondrial oxidation; STING agonism reduced acute and chronic colitis, and STING-expressing CD4+ T cells were increased in intestinal samples from patients with IBD. In separate ex vivo experiments, STING agonism increased IL-10 production by human CD4+ T cells (18). Other non-IBD work indicates that strong STING activation can induce T-cell stress or death, while a clinically relevant agonist can restrain a human Th17 inflammatory profile (46). These outcomes are stimulus- and dose-dependent and should not be collapsed into a single adaptive effect.

Treg biology also includes direct and indirect components. ILC3-dependent induction of microbiota-specific Tregs is supported by intestinal studies (28, 29), whereas dietary nucleic-acid sensing and antigen-presenting-cell cytokines provide broader regulatory routes (47). The available evidence therefore supports distinct T-cell-intrinsic, antigen-presenting-cell-mediated, and ILC3-mediated programs; it does not justify assigning all adaptive immune remodeling to one STING-associated network.

### 3.5 ILC3s: microbial sensing, tolerance, and activation-induced loss

ILC3s prevent the review from collapsing into a simple anti-STING argument (28). These cells support barrier integrity, microbial containment, and local immune tolerance. They can participate in microbiota-specific regulatory T-cell responses, making them relevant to the boundary between host defense and immune restraint (29).

In ILC3s, STING does not necessarily behave like the classic myeloid inflammatory amplifier described above. Under some conditions, intrinsic STING activity is linked to CCR7-dependent migration and the induction of microbiota-specific Tregs. This places STING within a tolerance-supporting program rather than a purely injury-promoting one.

The protective interpretation has a defined experimental limit. Inflammation-associated STING hyperactivation induced ILC3 death in the direct STING study (28). This supports activation-induced loss of a protective population, not a generalized or clinically established state of ILC3 “exhaustion”. Whether a comparable threshold operates in human IBD remains unresolved.

ILC3s therefore show why the STING paradox cannot be solved by assigning the pathway a single valence. Dose, duration, and tissue state matter. A pathway that helps coordinate microbial tolerance can become harmful when inflammatory stress is sustained and the cell type carrying the signal is no longer able to preserve its homeostatic program.

### 3.6 Intestinal epithelial cells: between barrier defense and tissue injury

Epithelial DNA sensing is positioned at the boundary between defense and injury (37). Controlled protective effects arise through more than one mechanism. cGAS can bind Beclin-1 and sustain epithelial autophagic flux without requiring a stronger canonical STING–interferon output; cGAS deficiency worsens epithelial injury in experimental colitis (19). In a separate human tissue and organoid study, inflamed IBD mucosa showed increased activated STING, and pharmacological STING activation in human colonic organoids enhanced release of mature MUC2 without increasing MUC2 transcription (20). This finding provides human-proximal evidence for a functional epithelial output, but it does not establish whether increased mucus release is protective, compensatory, or maladaptive *in vivo*. Epithelial STING can also promote REG3γ-dependent antimicrobial defense during enteric infection (38), while serine availability and redox control support cGAS–STING-dependent antiviral responses in the gut (39).

The direction of the epithelial response depends on variables that should be reported explicitly: agonist dose and duration; epithelial lineage and differentiation state; autophagy competence; serine and redox metabolism; mitochondrial or nuclear DNA release; microbial or viral exposure; and the dominant death pathway, including apoptosis, necroptosis, or inflammasome-associated pyroptosis. Disease model also matters. Infection challenges reveal antimicrobial functions, whereas DSS and 2,4,6-trinitrobenzene sulfonic acid (TNBS) emphasize chemically induced mucosal injury and repair. A protective or pathogenic conclusion should therefore be restricted to the tested combination of pathway node, epithelial state, exposure, and time window.

Intestinal stem cells add a further compartment. DHX9 loss causes R-loop accumulation, genomic instability, cGAS–STING activation, and impaired stem-cell function in experimental colitis (16). This mechanism is distinct from nuclear STING1–AHR regulation and shows how DNA damage can convert a repair compartment into a source of inflammatory signaling. Epithelial timing and injury burden are therefore important, but the direction of effect cannot be inferred from STING expression alone.

### 3.7 Multicellular integration determines the net STING effect

The net effect of STING in IBD is produced by interactions among several compartments. Myeloid cells often provide inflammatory amplification, dendritic cells connect damage sensing to adaptive skewing, ILC3s highlight tolerance and threshold effects, and epithelial cells connect barrier defense with injury propagation. These roles are not mutually exclusive, and they may coexist in the same patient at different sites of the gut.

A more cautious interpretation of the STING paradox is therefore needed. Cell type helps explain conflicting findings, but it does not fully resolve them unless disease activity, microbial state, tissue damage, and treatment exposure are also considered. For therapeutic design, the key question is not whether STING should be blocked or activated in general (42). It is which compartment is driving the lesion at the time intervention is considered.

## 4 Lesion state modifies STING output

### 4.1 The lesion-state framework is a working model, not a validated temporal sequence

The framework of barrier surveillance and early stress, active inflammation, and chronic or resolving lesions is used here to generate hypotheses rather than to represent an experimentally validated temporal sequence. Human IBD is relapsing, patchy, and treatment-shaped; epithelial injury, repair, microbial imbalance, myeloid activation, and regulatory feedback can coexist within the same patient and, in some cases, within different regions of the same biopsy specimen (14, 68). To our knowledge, no study has longitudinally followed all of the proposed STING states within the same IBD model or patient cohort.

The framework nevertheless permits comparison of experiments that emphasize different lesion states. Infection and homeostasis models reveal epithelial antimicrobial defense, cGAS-dependent autophagy, T-cell IL-10 programs, ILC3-mediated tolerance, and metabolically supported antiviral defense (18, 19, 28, 38, 39). Acute DSS or TNBS models emphasize epithelial damage and myeloid inflammatory amplification (21, 34–36). Chronic or resolution-focused studies address residual danger signals, reparatory macrophage transitions, and incomplete mucosal healing (42, 69, 70). These studies support individual components of the framework, but they do not establish a fixed transition from a protective state to pathogenic activation and then to renewed protection.

Accordingly, **Figure 3** maps evidence across lesion states rather than depicting a clinical timeline. The categories describe dominant programs that can overlap spatially and temporally. Therapeutic inference must therefore be anchored to current lesion activity, cell type, and direct evidence of target engagement rather than to a presumed disease phase.

**Figure 3:**
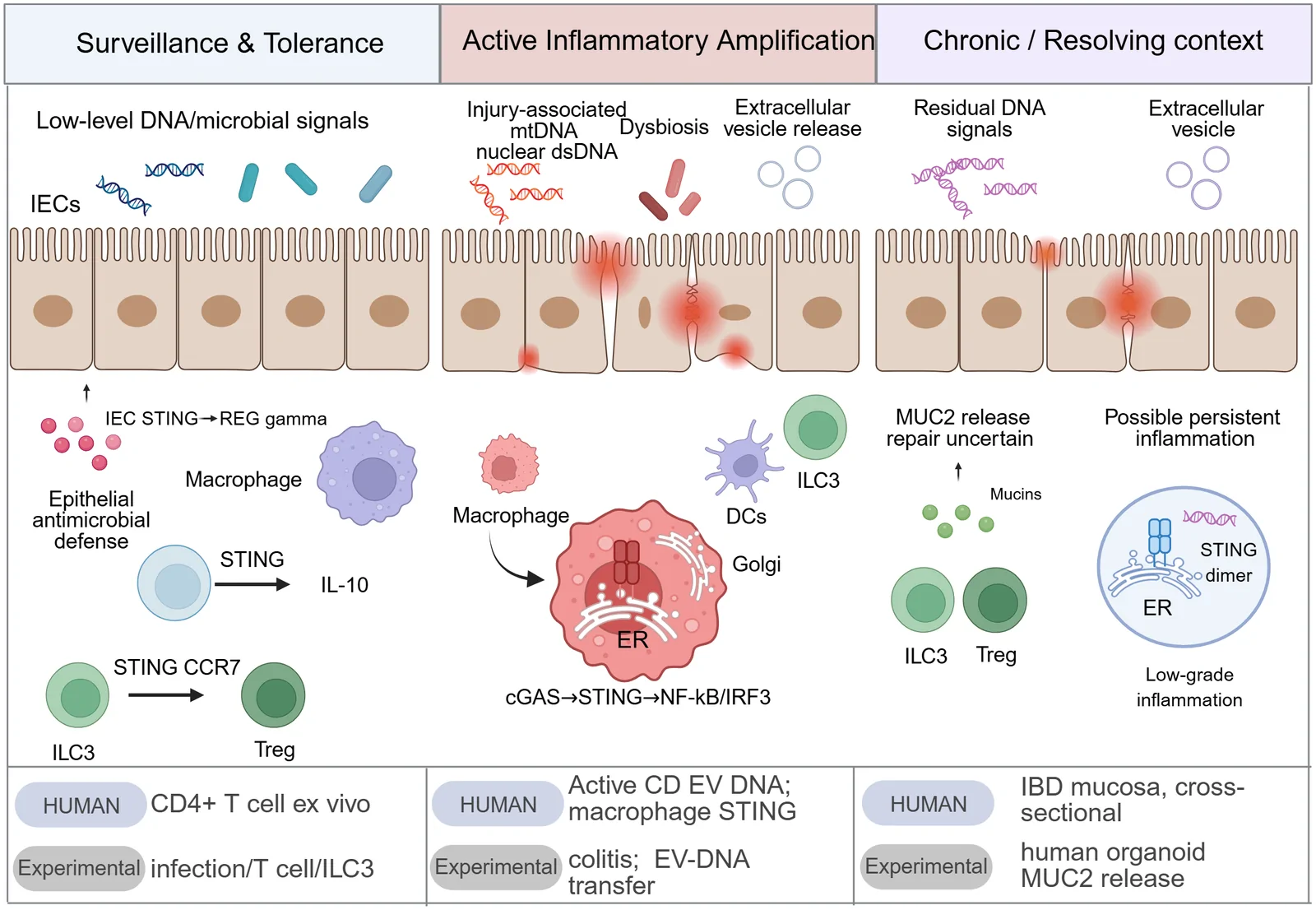
Evidence map across lesion contexts. The diagram organizes STING-related biology into surveillance/tolerance, active inflammatory amplification, and chronic/resolving lesion contexts. These categories can coexist and do not represent a validated clinical sequence. In active Crohn’s disease, increased extracellular-vesicle dsDNA is a human observation, whereas EV-induced macrophage STING activation is supported mainly by mechanistic assays. Recurrent or low-grade STING activity in chronic or resolving lesions remains a hypothesis requiring longitudinal target-engagement data. Human IBD mucosa and human colonic organoids provide direct evidence that STING activation can enhance mature MUC2 secretion (20), but the *in vivo* consequence remains uncertain. Created in BioRender. Bian, H. (2026) https://BioRender.com/nowfk0l.

### 4.2 Early barrier surveillance, stress, and tolerance maintenance

Initiation is the hardest phase to define in human IBD, because patients are usually sampled after inflammation is already clinically visible. Mechanistically, however, early disease can be modeled as a period in which barrier stress, microbial fluctuation, and limited danger-signal exposure begin to exceed normal mucosal buffering capacity (71). In that context, STING activation may contribute to pathogen restriction, sensing of DNA danger signals, and maintenance of epithelial–immune homeostasis (89).

Protective functions in early or low-burden settings are supported by several distinct systems: epithelial STING promotes antimicrobial REG3γ expression during enteric infection (38); T-cell-intrinsic STING can restrain pathogenic Th1 programs (18); and ILC3-intrinsic STING supports microbiota-specific tolerance (28). Commensal-triggered STING can also induce both inflammatory cytokines and IL-10 in mononuclear phagocytes, indicating that macrophage output is conditional rather than uniformly pathogenic (11). These findings support biological plausibility, but they do not establish a preclinical stage that can be identified prospectively in human IBD.

At the same time, early STING activation is not automatically beneficial. If DNA release is persistent, microbial exposure is high, or epithelial stress is not resolved, the same sensing program can lower the threshold for later inflammation. Initiation is therefore better described as a branching point than as a purely homeostatic phase.

### 4.3 Active inflammatory lesions: amplification dominates, but not every signal is equivalent

The strongest evidence for a pathogenic STING contribution in active lesions comes from experimental colitis models characterized by epithelial DNA release, dysbiosis, and heightened myeloid responsiveness (10, 21, 26, 34–36). Human active mucosa shows increased pathway components and interferon-related programs, but the available studies are predominantly cross-sectional and cannot determine whether STING initiates, amplifies, or merely accompanies the lesion (15, 17, 21).

The strongest pathogenic argument therefore concerns STING-dependent amplification within defined active-lesion models, not active IBD as a uniform human state. TNF-α, IL-1β, IL-6, IL-12, IL-23, type I interferons, and inflammasome-linked outputs may reinforce Th1/Th17 responses and epithelial injury, but each requires pathway-specific and cell-resolved evidence before being assigned to cGAS–STING.

Still, active disease should not be reduced to one pathway. STING is one component of a dense inflammatory network that also includes microbial receptors, cytokine loops, hypoxia, oxidative stress, and tissue-remodeling programs (72). The more rigorous conclusion is that STING can amplify active lesions when danger-signal burden and myeloid responsiveness are high, not that STING alone explains active IBD.

### 4.4 Chronic or resolving lesions: repair, residual danger signals, and inflammatory fixation

Chronic or resolving lesions are the settings in which the role of STING remains most uncertain. Clinical symptoms, endoscopic appearance, and histological activity do not necessarily resolve in parallel. Residual histological activity may persist despite clinical or endoscopic remission and is associated with a higher risk of relapse (69, 70). These discordant states make a simple protective-versus-pathogenic classification unreliable.

Resolution does not necessarily involve renewed protective STING activation. In a reparatory macrophage model, suppression of the Cmpk2–cGAS–STING pathway promoted macrophage transition toward a reparative state and facilitated resolution of intestinal inflammation (42). At the same time, protective STING programs described in T cells, ILC3s, and intestinal epithelial cells may remain relevant under lower-burden conditions. Whether these compartment-specific effects occur sequentially within the same colitis model has not been demonstrated. Renewed protection should therefore remain a hypothetical branch of the working model.

Persistent dysbiosis and mucosal pathway abnormalities can remain detectable during clinical or quiescent disease and may be associated with relapse (73, 74). These findings define a tissue environment in which recurrent danger signaling is biologically plausible, but they do not demonstrate persistent STING activation. Whether low-grade or repeated STING signaling maintains inflammatory responsiveness in myeloid or epithelial compartments remains a hypothesis requiring longitudinal, cell-resolved evidence.

### 4.5 Therapeutic timing follows from lesion state, compartment, and pathway engagement

The therapeutic implication is not that STING should be inhibited or activated according to a fixed disease-stage rule (75). A more defensible framework first identifies the dominant lesion program and the cellular compartment in which functional STING activation has been demonstrated (**Figure 4**). In a myeloid-rich active lesion with a high danger-signal burden, local or macrophage-directed inhibition may warrant preclinical evaluation. By contrast, broad suppression may be harmful when epithelial antimicrobial defense or ILC3-mediated tolerance remains necessary. This framework remains hypothetical because no prospectively validated biomarker currently identifies patients who are likely to benefit from STING inhibition or agonism.

**Figure 4:**
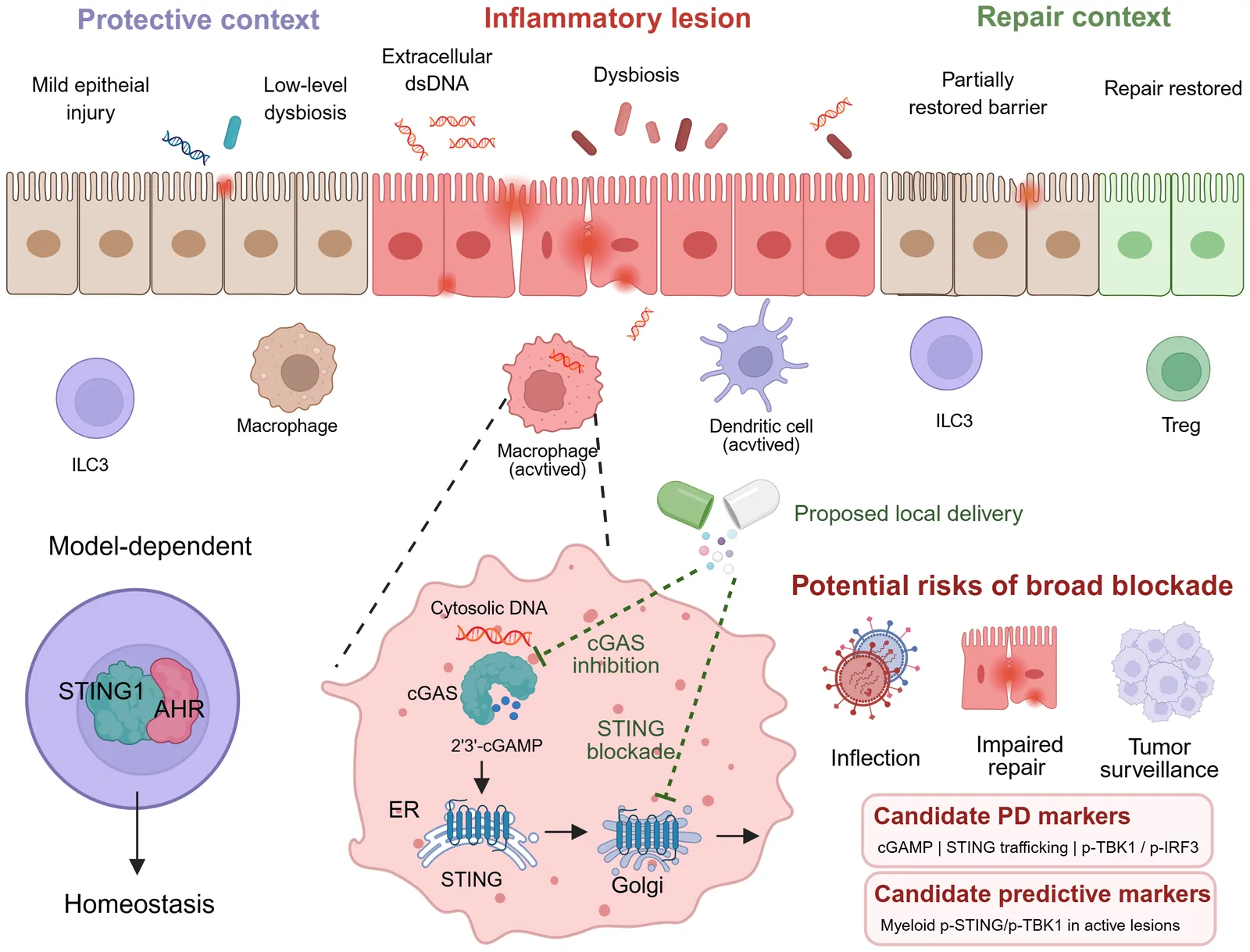
Context-dependent framework for STING-directed intervention. The schematic positions STING-directed therapy within protective, inflammatory and repair contexts. Nuclear STING1–AHR signaling is shown as a model-dependent homeostatic program, whereas cytosolic DNA–cGAS–2′3′-cGAMP–STING signaling in inflammatory myeloid cells represents the principal candidate target. Proposed approaches include cGAS inhibition, STING blockade and local delivery. Broad inhibition could compromise antimicrobial or antiviral defense, epithelial repair and tumor surveillance; intervention should therefore be restricted to lesions with demonstrable pathway activity and appropriate cellular localization. Candidate pharmacodynamic markers are cGAMP, STING trafficking, p-TBK1 and p-IRF3; myeloid p-STING/p-TBK1 in active lesions is presented as a candidate predictive marker. The framework is conceptual and not clinically validated. Created in BioRender. Bian, H. (2026) https://BioRender.com/igbbve5.

## 5 Evidence hierarchy: linking STING activity to therapeutic interpretation

The preceding sections conceptualize STING output as a mucosal program shaped by signal source, subcellular location, cell type, intensity, and lesion state. Evidence must therefore be appraised on two axes that answer different questions. Evidence directness asks whether a claim comes from human IBD, causal experimental colitis, an intestinal mechanistic system, or a non-IBD extrapolation. Activation depth asks whether the study measured abundance, cGAMP production, STING localization or phosphorylation, downstream signaling, or a target-linked biological outcome. **Table 1** keeps these axes separate and states how confidence was assigned.

Direct human IBD studies support disease relevance and localization but are usually cross-sectional and do not prove causality. Cell-specific genetic or pharmacological perturbation in experimental colitis can support causality within the tested model, whereas intestinal organoids and primary cells isolate mechanism at the cost of tissue ecology. Non-IBD inflammatory or autoimmune studies provide extrapolated mechanistic evidence only. Within every source category, increased cGAS, STING, or interferon-stimulated gene expression remains an abundance readout; it does not establish cGAMP production, STING trafficking or phosphorylation, TBK1–IRF3 or NF-κB activation, type I interferon output, or a functional biological consequence.

### 5.1 Human mucosal evidence supports relevance, localization, and stratification

Human tissue evidence is the necessary starting point because it places STING biology in the diseased mucosa rather than in an abstract pathway diagram. Transcriptomic, single-cell, and spatial studies of IBD mucosa have identified epithelial injury programs, activated myeloid states, stromal remodeling, T-cell activation, and interferon-related inflammatory niches (22, 23). More focused analyses suggest that cGAS-STING components or adjacent transcriptional programs are enriched in subsets of ulcerative colitis or Crohn’s disease tissue. These observations justify taking the pathway seriously in human IBD, but they do not by themselves define therapeutic direction. Human mucosal ISG15 expression further illustrates why an interferon-related signature should be treated as evidence of pathway-associated inflammation rather than proof of functional STING activation (24).

The strength of human mucosal profiling is disease relevance; its weaknesses are causal ambiguity and incomplete stratification. Whole-tissue cGAS/STING abundance may reflect epithelial stress, infiltration by STING-expressing myeloid cells, altered cell composition, or defective pathway termination. Functional activation should therefore be supported, where possible, by cGAMP measurement, STING redistribution or phosphorylation, phospho-TBK1/phospho-IRF3, NF-κB readouts, type I interferon output, and a linked cellular or tissue phenotype. **Table 2** summarizes the available human studies and reports disease subtype, location, activity, treatment status, tissue, cell type, and readout when these data were available.

**Table 2:** Selected human studies relevant to cGAS–STING signaling in UC and CD.

Study	Cohort/phenotype	Treatment status	Sample	STING-related readout	Resolution	Main finding/limit
Smillie et al. (14)	18 UC; 12 healthy; 366,650 cells	Not reported	Colonic mucosa	Single-cell transcriptomes	Cell-resolved	Maps UC cell states; no biochemical assay of STING activation
Wang et al. (15)	GSE38713: 15 active UC/13 healthy; GSE47908: 39 UC/15 healthy; local IHC: 21 UC before infliximab	Before infliximab (local IHC cohort)	Colon	cGAS–STING expression/subtyping; IFI16 IHC	Bulk + IHC	Associative stratification; expression does not establish pathway activation
Zhao et al. (17)	Active CD: 5; non-CD controls: 5	Not reported	Plasma EVs; macrophage assays	EV dsDNA; STING-dependent macrophage response	Ex vivo macrophage	Human association; causality rests mainly on experimental systems
Chen et al. (21)	15 active UC; 9 remission UC; 45 controls; immunoblot: 6/group	Not reported	Colonic tissue/biopsy	cGAS/STING/TBK1/IRF3 and phosphorylated forms	Bulk tissue	Activity-linked signaling; cell source and treatment confounding remain
Ren et al. (16)	UC: 5/4 controls; CD: 6/5 controls	Not reported	Intestinal biopsy	DHX9 protein	Bulk tissue; mouse ISC mechanism	DHX9 is reduced in IBD; cGAS–STING causality derives from epithelial mouse models
Yang et al. (18)	Colonic biopsies: 12 healthy/7 UC; separate tissue-array and PBMC experiments	Not reported	Colon; blood-derived T cells	STING+ CD4+ cells; cGAMP-induced IL-10	T-cell-resolved/ex vivo	Supports a protective T-cell program, not global mucosal activation
Khan et al. (19)	UC/controls: mRNA 8/group; immunoblot 7/group; IF 4/group	Not reported	Colonic epithelium	cGAS abundance; Beclin-1/autophagy tested in mice and IECs	Epithelial	Human upregulation is associative; homeostatic causality is preclinical and partly STING-independent
Yang et al. (20)	Inflamed and non-inflamed ileal/colonic mucosal transcriptomes (821 samples) with histological validation; human colonic organoids	Not reported for the source cohorts	IBD ileum and colon; human crypt-derived colonic organoids	Mucin and cGAS–STING expression; p-STING; diABZI/H-151 perturbation; mature and immature MUC2	Human tissue + organoid functional assay	Links epithelial STING activation to mature MUC2 secretion; observational tissue data and pharmacological organoid evidence do not establish benefit or harm *in vivo*

### 5.2 Human UC and CD data should not be pooled uncritically

Available studies do not establish a reproducible difference in cGAS–STING activation between ulcerative colitis (UC) and Crohn’s disease (CD). UC studies predominantly sample inflamed colonic mucosa, where bulk epithelial injury and mucosal myeloid infiltration can elevate pathway-associated readouts (15, 21). CD studies include blood-derived extracellular vesicles and ileal or colonic tissue, but are fewer and heterogeneous (17, 23). Direct head-to-head analyses with matched activity, treatment exposure, and anatomical site are lacking.

Stratification is nevertheless biologically necessary. UC primarily affects the colon and is characterized predominantly by mucosal inflammation, whereas CD can involve different regions of the gastrointestinal tract and may extend through the bowel wall. These anatomical and histological differences influence epithelial injury, microbial ecology, immune-cell composition, and the exposure of responsive compartments to luminal or circulating DNA. An apparent difference between UC and CD may therefore reflect tissue location, disease activity, medication exposure, or cellular composition rather than diagnosis alone. Claims should be stratified by UC versus CD, colonic versus ileal disease, and active versus inactive tissue whenever the source study permits. Where such information is unavailable, the resulting uncertainty should be stated explicitly.

### 5.3 Preclinical models support mechanism, but model context shapes the conclusion

Experimental models remain essential because they allow causal testing. Genetic deletion, pharmacological inhibition, agonist challenge, microbial manipulation, epithelial injury, and cell-type-specific systems can ask whether STING contributes to a defined disease process. These approaches provide much of the mechanistic evidence linking epithelial damage-derived DNA, dysbiosis, mitochondrial stress, and myeloid STING activation to inflammatory amplification.

Each model, however, reproduces only selected features of mucosal disease. DSS colitis is particularly informative for barrier disruption and injury-driven innate inflammation, whereas TNBS colitis models hapten-associated immune activation. Neither fully reproduces the chronic, relapsing, treatment-modified, and anatomically heterogeneous course of human IBD (76, 77). These models may preferentially reveal settings in which STING inhibition reduces injury, particularly during active inflammation with a high danger-signal burden and marked myeloid activation. A therapeutic inference becomes more robust when the direction of effect is reproduced across models representing distinct lesion states, including chronic, resolving, microbiota-dependent, epithelial-restricted, and immune-cell-specific systems (68).

### 5.4 Cell-resolved evidence is required for therapeutic interpretation

Therapeutic interpretation requires identification of a harmful STING output within a defined mucosal compartment. Myeloid cells currently provide some of the strongest experimental evidence for STING-dependent inflammatory amplification during active intestinal injury, although the magnitude and direction of the effect vary across models. In selected experimental systems, dendritic-cell STING has been implicated in linking tissue-derived danger signals to cytokine-mediated adaptive immune responses.

These distinctions make single pathway readouts difficult to interpret. An interferon-related signature in inflamed tissue may reflect antiviral defense, pathogenic myeloid activation, epithelial stress, treatment-related immune remodeling, or several overlapping programs. Increased STING expression may mean stronger pathway activation, greater infiltration of STING-expressing cells, or defective pathway termination. Conversely, a low whole-tissue signal may miss focal activation in a small but biologically important compartment.

A useful evidence hierarchy separates five levels: (i) abundance of cGAS, STING, or related transcripts and proteins; (ii) cGAMP production; (iii) STING trafficking and phosphorylation; (iv) downstream TBK1–IRF3, NF-κB, and type I interferon outputs; and (v) cellular or tissue consequences. Cellular and subcellular localization must be reported across these levels. Therapeutic direction depends most strongly on target engagement linked to a functional consequence, not on abundance alone.

### 5.5 The current evidence supports testable rules, not routine global inhibition

Taken together, the available evidence supports a focused translational hypothesis: STING may be targetable in selected IBD lesions where DNA danger signals, microbial imbalance, and myeloid inflammatory programs converge. It does not yet support routine global STING inhibition as a general IBD strategy. A pathway that can defend the barrier, sense microbial invasion, promote tolerance, amplify inflammation, and worsen tissue injury cannot be treated as uniformly harmful.

This evidence can be translated into a testable framework, but not yet into clinically validated selection criteria. Evidence of relevant pathway engagement should be based on convergent measurements, such as cGAMP abundance, STING redistribution or phosphorylation, downstream signaling, and localization to the implicated cellular compartment, rather than on cGAS or STING expression alone. Lesion state should also be defined because active injury with myeloid amplification differs biologically from resolution, mucosal repair, or low-grade chronic inflammation. Cellular localization should guide therapeutic reasoning: inhibition may be more plausible when pathogenic output is concentrated in macrophages or dendritic cells, whereas broad suppression may carry greater risk when epithelial defense, microbial containment, or ILC3-associated immune regulation remains important. These considerations favor evaluation of local, time-limited, or compartment-biased modulation rather than prolonged systemic pathway suppression. Their clinical value, however, remains to be established prospectively.

## 6 Therapeutic windows for STING-directed intervention

### 6.1 From pathway inhibition to defining a therapeutic window

STING is an attractive therapeutic target because it links DNA sensing to several processes relevant to IBD, including epithelial injury responses, microbiota-associated stimulation, and mucosal immune activation (4). That position creates opportunity and risk in equal measure. Blocking the pathway may interrupt inflammatory amplification, but the same intervention could weaken host defense, tolerance induction, epithelial repair, or resolution. This therapeutic tension is shared across inflammatory diseases and reinforces the need to distinguish molecular target engagement from downstream clinical benefit (78).

The practical question is how to identify lesions in which a pathogenic STING output is dominant and pharmacologically accessible. This requires three linked decisions: whether functional pathway activation is present, which cellular compartment sustains it, and whether suppression of that activity is compatible with the lesion’s requirements for host defense and tissue repair. These are proposed, testable treatment contexts rather than clinically validated selection criteria.

A pharmacologically informative evaluation should therefore specify the molecular target, intervention timing, model, route, cell selectivity, target-engagement evidence, efficacy, and anticipated effects on antimicrobial defense and mucosal repair. **Table 3** applies these criteria to representative modulators. Reduction of an acute colitis score is informative, but does not by itself establish suitability for chronic human IBD.

**Table 3:** Representative cGAS–STING modulators and translational constraints.

Agent/strategy	Mechanism	Model/route	Engagement and efficacy	Key limitation/status
RU.521 micelles (36, 52)	Direct cGAS catalytic-site inhibition; lowers cGAMP	Murine colitis and macrophages; oral inflammation-responsive delivery; preventive/therapeutic schedules	Reduced pathway readouts, disease activity and tissue inflammation	Preclinical IBD; site-biased delivery is not cell selectivity; chronic infection, repair and systemic exposure unresolved
Low-dose ganciclovir (34)	Indirect suppression of macrophage cGAS–STING; direct binding unshown	DSS colitis and macrophages; intraperitoneal 10 mg/kg/day during DSS; STING-deficient mice	STING dependence; reduced colitis, pain and dysbiosis	Repurposed antiviral; precise target, off-target effects and chronic safety unresolved
H-151/C-176 (53)	Covalent binding to STING Cys91; blocks palmitoylation	Cells and non-IBD autoinflammatory mice; systemic dosing	Direct STING engagement; reduced inflammation outside IBD	No IBD efficacy or intestinal selectivity; host-defense risk; preclinical
SN-011 (54)	Occupies the STING cyclic-dinucleotide-binding pocket	Cellular and non-IBD autoimmune models; systemic dosing	Direct pocket occupancy; suppressed STING-driven inflammation outside IBD	No IBD efficacy, cell selectivity or long-term mucosal safety data
UNC9036/STING PROTACs (55)	VHL-recruiting degradation of activated/phospho-STING	Renal cell lines and antiviral assays; *in vitro* proof of concept	STING depletion and reduced p-IRF3; suppressed innate and antiviral signaling	No colitis data; transient agonism, antiviral effects and delivery remain concerns; discovery stage
Trafficking/termination modulation	ER–Golgi transport, autophagic/lysosomal clearance or phase separation	Mechanistic cell studies; no established IBD regimen	Requires node-specific assays; cytokine reduction alone cannot identify the inhibited step	IBD efficacy unknown; may disrupt protective signaling and signal termination

### 6.2 Preclinical strategies for modulating cGAS-STING signaling

cGAS inhibition and STING inhibition are pharmacologically distinct. RU.521 binds within the catalytic pocket of cGAS and inhibits cGAMP synthesis; it is not a direct STING inhibitor (36, 52). Direct STING antagonists include compounds that occupy the cyclic-dinucleotide-binding pocket, such as SN-011, and covalent inhibitors such as H-151 or C-176 that block palmitoylation-dependent signaling at the Golgi (53, 54). These mechanisms differ from interventions that alter trafficking or induce STING degradation.

The most disease-proximal pharmacological evidence remains preclinical. Inflammation-responsive micelles delivering RU.521 reduced experimental colitis and provided site-biased cGAS inhibition (36). Low-dose ganciclovir suppressed macrophage cGAS–STING readouts and improved DSS colitis in a STING-dependent manner, but its precise molecular interaction with the pathway remains indirect (34). H-151/C-176 and SN-011 establish direct STING druggability in non-IBD inflammatory models; their efficacy, dosing, and safety in IBD remain untested (53, 54).

Targeted STING degradation is an additional proof-of-concept modality. VHL-recruiting STING proteolysis-targeting chimeras, including UNC9036, reduce STING abundance and downstream IRF3 signaling in renal carcinoma cell lines. Because these degraders are derived from a STING agonist, transient STING activation and attenuation of antiviral responses have been observed *in vitro*, whereas intestinal delivery and efficacy in IBD remain unresolved (55). Trafficking-directed approaches are likewise mechanistically attractive but have not yet established selective therapeutic benefit in colitis. These categories should therefore be presented as distinct experimental strategies rather than as interchangeable “STING inhibitors”.

Collectively, these studies establish druggability more clearly than clinical utility. Most lack human IBD target-engagement data, long-term exposure, and cell-selective delivery. Their consequences may differ substantially: cGAS inhibition preserves cGAS-independent STING inputs; ligand-pocket blockade and palmitoylation inhibition act at different STING states; trafficking interventions may alter termination as well as activation; and degradation removes scaffold and signaling functions together.

STING regulation includes oligomerization, ER-to-Golgi trafficking, palmitoylation, autophagic or lysosomal turnover, proteasomal degradation, and phase separation (55, 56, 59, 79). Chronic mucosal disease may reflect not only excessive activation but also failed termination. Interventions aimed at these steps require direct target-engagement assays because reduced cytokine output alone does not identify the inhibited node.

Delivery is not a technical afterthought here. It is part of the biological argument (80). Because IBD lesions are mucosal and often segmental, oral local-release systems or inflammation-responsive delivery may be necessary to keep the intervention near the pathogenic compartment and away from systemic STING functions (81). Lipid- and nanoparticle-based delivery has provided proof of concept in experimental colitis, but cell-specific target engagement, efficacy in chronic disease, and long-term safety remain to be established (82).

The goal of optimized delivery is not only to bring a drug into the intestine. It is to bias intervention toward the cell types in which STING has become pathologically amplified. During active IBD, the most relevant inhibitory targets are likely myeloid cells and damaged epithelium rather than all STING-expressing cells. Combining local administration, nanomaterials, and cell-targeting approaches may improve benefit by sparing homeostatic STING functions.

### 6.3 Translational barriers to context-guided STING modulation

The first barrier is cell-type divergence. Inhibiting macrophage- or dendritic-cell-mediated inflammatory amplification may be beneficial in selected active lesions, whereas the same exposure could disrupt epithelial REG3γ-associated defense, reported T-cell IL-10 responses, direct STING-dependent functions in ILC3s, or nuclear STING1–AHR homeostatic programs (18, 25, 28, 38). Local or cell-biased delivery is therefore part of the biological rationale, not merely a formulation preference.

A second barrier is the mismatch between acute models and chronic clinical use. Systemic or prolonged suppression could increase susceptibility to bacterial or viral infection, weaken type I interferon-dependent antiviral immunity, impair mucosal healing, or alter tumor immune surveillance. These risks are particularly relevant in colitis-associated colorectal cancer, where STING may contribute to antitumor immunity as well as inflammation (83). Long-term safety, infection challenge, wound healing, and tumor-surveillance endpoints should accompany efficacy studies.

Most evidence regarding STING in IBD still comes from mouse models and *in vitro* systems, whereas longitudinal human mucosal data and prospective target-engagement studies remain limited (13). As of the literature-search cutoff, no interventional clinical trial has evaluated a cGAS or STING modulator as a treatment for IBD. Human evidence currently consists mainly of molecular pathology, ex vivo assays, and multi-omics analyses of patient samples; these findings establish relevance but not clinical efficacy or a validated selection strategy.

### 6.4 Future directions: biomarkers, patient selection, and local delivery

Patient selection requires measurable biomarkers rather than broad labels such as “mucosal STING activity”, “DNA damage”, “IFN”, or “myeloid signature”. Candidate pharmacodynamic biomarkers include tissue cGAMP, STING relocalization from ER to Golgi, phospho-STING, phospho-TBK1, phospho-IRF3, and a short-interval change in pathway-linked cytokines after treatment. These readouts indicate target engagement but do not, on their own, predict benefit.

Candidate predictive biomarkers should enrich for lesions in which a pathogenic STING output is likely to contribute materially to disease activity: cell-resolved phospho-STING/TBK1 activity in inflammatory macrophages or dendritic cells, extracellular-vesicle DNA or cytosolic DNA burden linked to those cells, epithelial injury with demonstrable cGAS–STING activation, and spatial co-localization of pathway activity with histologically active disease. Conversely, preserved epithelial antimicrobial programs, reported T-cell IL-10 responses, and direct STING-dependent homeostatic functions in ILC3s may argue against broad pathway inhibition. A high risk of infection should be treated as a clinical safety modifier rather than as a predictive biomarker of treatment response. These remain candidate biomarkers and require prospective validation. Existing single-cell and molecular-classification studies provide a framework for patient stratification, but none has validated a STING-specific predictive biomarker (84–88).

Single-cell and spatial methods can assign these biomarkers to defined cells and lesion sites, but transcriptional signatures should be paired with protein localization, phosphorylation, metabolite measurement, or perturbation whenever possible. Whole-tissue upregulation is insufficient because it cannot distinguish increased expression per cell from altered cell composition or focal activation in a small compartment.

Validation should proceed through complementary experiments rather than a single model. Priorities include cell-specific cGAS or STING deletion; dose- and time-response studies in acute, chronic, and resolving colitis; microbiota-dependent and infection-challenge models; intestinal organoid–immune-cell co-culture; comparison of local with systemic inhibition; and long-term assessment of healing, antiviral defense, and tumor surveillance. In parallel, longitudinal paired biopsies from ileal and colonic lesions should combine spatial or single-cell profiling with biochemical target-engagement assays and treatment exposure.

These experiments should test the working model rather than presuppose it. They should determine whether the same lesion moves between protective and pathogenic STING states, whether subcellular localization predicts output, and whether cell-biased modulation improves efficacy without disabling protective compartments. Until such evidence is available, terms such as “best-fit context” and “selection marker” should be read as proposed testable contexts and candidate biomarkers, not as clinically established criteria.

## 7 Discussion

The strongest mechanistic evidence in this field is causal but predominantly preclinical. Cell-specific perturbation and pharmacological studies show that cGAS–STING can amplify inflammation in selected injury-associated myeloid and epithelial contexts. Other models provide varying levels of evidence for antimicrobial defense, direct T-cell-intrinsic regulation, STING-dependent microbial sensing by ILC3s, and nuclear STING1–AHR activity (10, 18, 21, 25, 26, 28, 35, 38, 64). These protective or homeostatic functions should not be assigned equivalent certainty, and broader ILC3-mediated tolerance should not be assumed to be STING dependent without direct perturbation evidence. The principal evidence gap lies in translating these model-dependent findings into a biologically defined human treatment population. Human studies support disease association and anatomical or cellular localization, but are generally limited in scale, predominantly cross-sectional, and inconsistently stratified by diagnosis, anatomical site, disease activity, and treatment exposure.

Several contradictions therefore remain unresolved. Increased cGAS or STING expression may accompany functional pathway activation, but it may also reflect a response to tissue damage, altered cellular composition, or impaired pathway termination. Opposing STING effects across infection, immune-tolerance, and chemically induced colitis models may reflect differences in signal source, magnitude and duration, cellular compartment, and tissue state rather than a temporally ordered switch within the same lesion. Likewise, an interferon-related signature can arise from cGAS–STING, RNA sensing, or other inflammatory pathways. Distinguishing among these interpretations requires cell-resolved measurements of pathway activation together with causal perturbation; pharmacological studies additionally require direct evidence of target engagement.

This review also has limitations. This article is a structured narrative synthesis rather than a systematic review. Heterogeneity in study design, experimental systems, and reported outcomes precluded formal quantitative comparison. Several recently proposed mechanisms are supported primarily by non-IBD settings or individual experimental models, and evidence for UC–CD differences, longitudinal transitions between lesion states, and the safety of chronic pharmacological modulation remains limited. We therefore present the compartment- and lesion-state framework as a working model. Progress toward clinical translation will require longitudinal validation in human tissue, cell-resolved biomarkers, evaluation of local or cell-biased delivery, and explicit assessment of infection susceptibility, mucosal repair, and tumor-surveillance risks.
